# Improved Human Bone Marrow Mesenchymal Stem Cell Osteogenesis in 3D Bioprinted Tissue Scaffolds with Low Intensity Pulsed Ultrasound Stimulation

**DOI:** 10.1038/srep32876

**Published:** 2016-09-06

**Authors:** Xuan Zhou, Nathan J. Castro, Wei Zhu, Haitao Cui, Mitra Aliabouzar, Kausik Sarkar, Lijie Grace Zhang

**Affiliations:** 1 Department of Mechanical and Aerospace Engineering, The George Washington University, Washington DC 20052, USA; 2 Department of Biomedical Engineering, The George Washington University, Washington DC 20052, USA; 3 Department of Medicine, The George Washington University, Washington DC 20052, USA

## Abstract

3D printing and ultrasound techniques are showing great promise in the evolution of human musculoskeletal tissue repair and regeneration medicine. The uniqueness of the present study was to combine low intensity pulsed ultrasound (LIPUS) and advanced 3D printing techniques to synergistically improve growth and osteogenic differentiation of human mesenchymal stem cells (MSC). Specifically, polyethylene glycol diacrylate bioinks containing cell adhesive Arginine-Glycine-Aspartic acid-Serene (RGDS) peptide and/or nanocrystalline hydroxyapatite (nHA) were used to fabricate 3D scaffolds with different geometric patterns via novel table-top stereolithography 3D printer. The resultant scaffolds provide a highly porous and interconnected 3D environment to support cell proliferation. Scaffolds with small square pores were determined to be the optimal geometric pattern for MSC attachment and growth. The optimal LIPUS working parameters were determined to be 1.5 MHz, 20% duty cycle with 150 mW/cm^2^ intensity. Results demonstrated that RGDS peptide and nHA containing 3D printed scaffolds under LIPUS treatment can greatly promote MSC proliferation, alkaline phosphatase activity, calcium deposition and total protein content. These results illustrate the effectiveness of the combination of LIPUS and biomimetic 3D printing scaffolds as a valuable combinatorial tool for improved MSC function, thus make them promising for future clinical and various regenerative medicine application.

Human bone marrow mesenchymal stem cell (MSC), a subset of adult multipotent stem cell populations residing within the bone stroma are one of the most important cells related to musculoskeletal tissue formation. MSC can terminally differentiate into a multitude of other mature cell types, including chondrocytes, myocytes, adipocytes and tenocytes amongst others^1^,^2^. Therefore, they have been widely investigated for a variety of tissue engineering and regenerative medicine applications. However, despite their remarkable potential, one of the major challenges in successfully applying stem cells in functional tissue regeneration is the difficulty in providing the proper environmental cues to regulate their self-renewal and differentiation^3^,^4^,^5^,^6^. In the human body, stem cells reside within a 3D extracellular matrix (ECM) with spatiotemporal chemical and physical cues. It is clear that engineering a biomimetic 3D tissue environment will facilitate deciphering the mechanisms of controlling stem cell behavior and have a profound impact on stem cell based tissue regeneration^7^. However, due to the complex nature of most human tissue, a significant challenge in directing stem cell function for tissue regeneration is to create the biomimetic 3D tissue environment with both hierarchical nano-micro-macro structure and spatially distributed bioactive components.

As an emerging complex tissue fabrication technique, 3D printing has shown great promise in fabricating clinically-relevant artificial tissue scaffolds with hierarchical micro to macro structure from versatile computer-aided designs (CAD)^8^. These scaffolds can be designed to closely match the geometry of a defect site allowing for increased host-implant interaction resulting in greater integration. More specifically, the 3D printing scaffold cannot only be designed with varying inner pore and channel structure, but also customized to fit a specific patient’s defect based on medical imaging data^9^. Moreover, our lab has successfully shown that 3D printed tissue scaffolds can be fabricated from a variety of nanomaterials in addition to the incorporation of growth factors to further influence the local environment of the injury^4^,^5^,^10^. With the development of new printing technologies and biomaterials, 3D printing is expected to play a critical role in future complextissue and organ regeneration^11^. A novel table-top stereolithography-based 3D bioprinter in our lab was based on photocrosslinking platform using rapid prototyping lithographic methods^12^. It is composed of a photocurable bioink, an X–Y control laser generator, and a Z-control movable stage. This 3D bioprinter works by adding one layer of material on top of previous solidified layer by a ‘layer-by-layer’ method. One of the advantages of stereolithography-based 3D bioprinters is the capability of employing various bioactive and biomimetic nanocomponents into the photocurable bioink solution for manufacturing multifunctional 3D nanocomposite scaffolds.

Nanocrystalline hydroxyapatite (nHA), is the primary mineral component in bone extracellular matrix, and has been widely used for numerous hard tissue (e.g., bone and teeth) regeneration due to its excellent biocompatibility and biomimicry. It has been shown that nHA induces osteogenesis differentiation of MSC and improve biomineralization when compared to many other implant materials^6^,^13^. In this study, we incorporated nHA into photocurable polyethylene (glycol) diacrylate (PEGDA) bioink for 3D printing biomimetic nanocomposite bone scaffolds. In addition, Arginine-Glycine-Aspartic acid-Serene (RGDS) peptide, as a common integrin binding sequence, has been widely used for promoting cell adhesion^14^,^15^. We utilized this tetrapeptide to improve cell adhesion of the 3D printed scaffold. Collectively, these diverse bioactive nanocomposites and molecules coupled with photocurable bioink can highly enhance the multifunctionality of 3D scaffold via our stereolithography-based 3D bioprinter.

Owing to the regenerative capacity of bone, this allows for most injuries to regenerate with few side effects, albeit very slowly without any exogenous factors or stimulation. The rate and extent of healing can theoretically be increased by incorporating physical or biochemical cues into the injury site. Several methods of promoting the healing of fractures have been proposed, including physical stress, electromagnetic field stimulation^16^, localized bioactive factor release (ie. bone morphogenic proteins), and pharmaceuticals (ie. alendronate)^17^ with some approved for use clinical use^18^. Ultrasound (US) therapy is an effective method of mechanical stimulation which has been widely applied for clinical diagnosis and therapeutic applications. Low-intensity pulsed ultrasound (LIPUS) has been reported to improve MSC differentiation, promoting bone union and facilitating bone fracture healing^19^. Toru Iwashina^19^ reported that LIPUS improved cell proliferation and proteoglycan production of rabbit intervertebral disc cells after 5–12 days treatment. Uddin SM^20^,^21^,^22^ found that LIPUS can be as a non-invasive acoustic tool for bone repairing, osteogenic differentiation and proliferation of MSCs/osteoblaset. Pilla^23^ also reported that LIPUS can significantly accelerate fibular bone repair in the rabbit body for 20 minutes daily after sustaining 28 days of stimulation. However, most reports of LIPUS has focused on 2D culture plates or hydrogel thin films^18^,^24^,^25^,^26^. There is lack of an efficient biomimetic 3D environment to investigate the LIPUS action on cell cluster and 3D tissue regeneration.

Therefore, the objective of the current work was to investigate the effectiveness of the combination of LIPUS and biomimetic 3D printing scaffolds as a valuable combinatorial tool for improved growth and osteogenic differentiation of MSC for future regenerative medicine applications. For this purpose, 3D scaffolds with different geometries pattern were printed and optimized by stereolithography-based 3D bioprinter as well as the optimal work parameters of LIPUS were evaluated. The physicochemical and biological characteristics of 3D scaffolds coupled with RGDS and nHA were examined. The 1, 3 and 5 day proliferation and three week osteogenic differentiation of MSC in 3D printed bone scaffolds under LIPUS treatment were thoroughly evaluated.

## Results and Discussion

### Synthesis and Characterization of 3D Printed Scaffold

**Figure 1:**
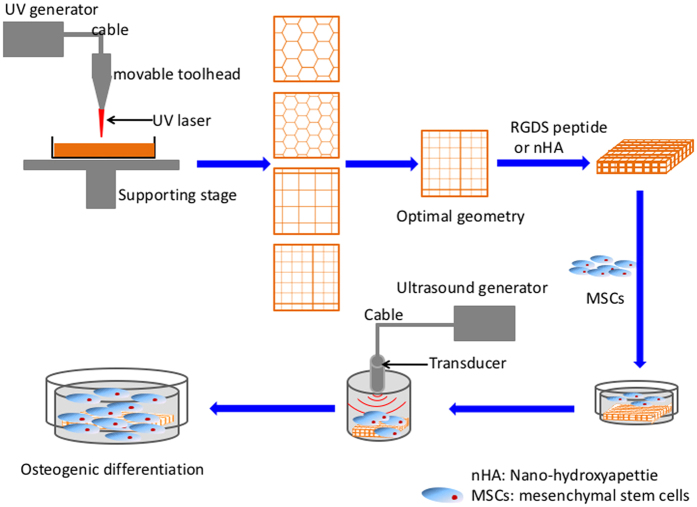
Schematic diagram of 3D printed scaffold fabrication and MSC excitation by low intensity pulsed ultrasound (LIPUS).

**Figure 2:**
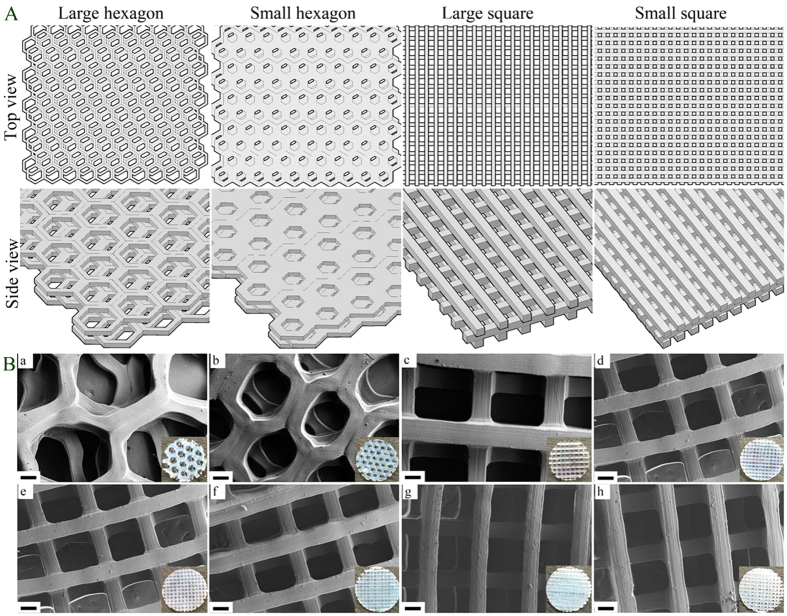
(**A**) CAD models of four bone scaffolds with large hexagonal (LH), small hexagonal (SH), large square (LS) and small square (SS) pore structure. (**B**) SEM images of various 3D printed scaffolds: (a) LH; (b) SH; (c) LS; and (d) SS pore structure. SEM images of small square pore scaffolds with or without RGDS and nHA: (e) PEGDA; (f) PEGDA-RGDS; (g) PEGDA- nHA; and (h) PEGDA-nHA-RGDS scaffolds. The insert images are the corresponding scaffolds’ photographs. Scale bar = 100 μm.

In this study, firstly, scaffolds with various geometries (small hexagonal (SH), large hexagonal (LH), small square (SS) and large square (LS)) were 3D printed via our table-top stereolithograghy apparatus (Fig. 1). Scanning electron microscope (SEM) morphology and Computer-aided design (CAD) models of scaffolds are shown in Fig. 2. All scaffold geometries correlated well with the CAD models (Fig. 2(a–d)) with distinct 3D structure. The small square pattern has more pores than the large one. The porosities of each 3D printed hydrogel scaffold were calculated as 64% (large square), 53.8% (small square), 78.5% (large hexagon) and 63.9% (small hexagon), respectively. The porosities increased with the increasing pore size in both square and hexagon patterns.

The morphology of 3D printed small square pore scaffolds with varied bioactive nanomaterials (i.e., nHA and RGDS peptide) was characterized by SEM (Fig. 2(e–h)). Scaffolds with PEGDA and PEGDA-RGDS exhibited a smooth surface while scaffolds with nHA (PEGDA-nHA and PEGDA-RGDS-nHA) show a matte surface. The pores and channels in all scaffolds were distinct and mirrored the pre-designed shape well. The morphology of scaffolds indicated that 3D printed scaffolds could be successfully prepared by our stereolithograghy-based 3D printer with different requirements.

The wettability of all scaffolds was evaluated by contact angle analyzer and the results are shown in Figure S1. The equilibrium contact angle reflects the relative molecular interaction at the three-phase boundary of liquid, solid and gas. Even though nHA and RGDS peptide were incorporated within the scaffold, no significant difference was observed.

Young’s modulus of all printed scaffolds was determined by ultimate tensile testing (Table 1). The results demonstrated that scaffolds with lower porosity possess higher mechanical properties than those of higher porosity, while further enhancement is observed with the presence of nHA which is known to increase material stiffness. Specifically, the Young’s modulus of small square PEGDA scaffolds exhibited a 100% increase when compared to large hexagon PEGDA scaffolds. A nearly 150% increase was observed amongst PEGDA-nHA and PEGDA-RGDS-nHA group.

### Determination of Optimal Geometric Pattern

**Figure 3:**
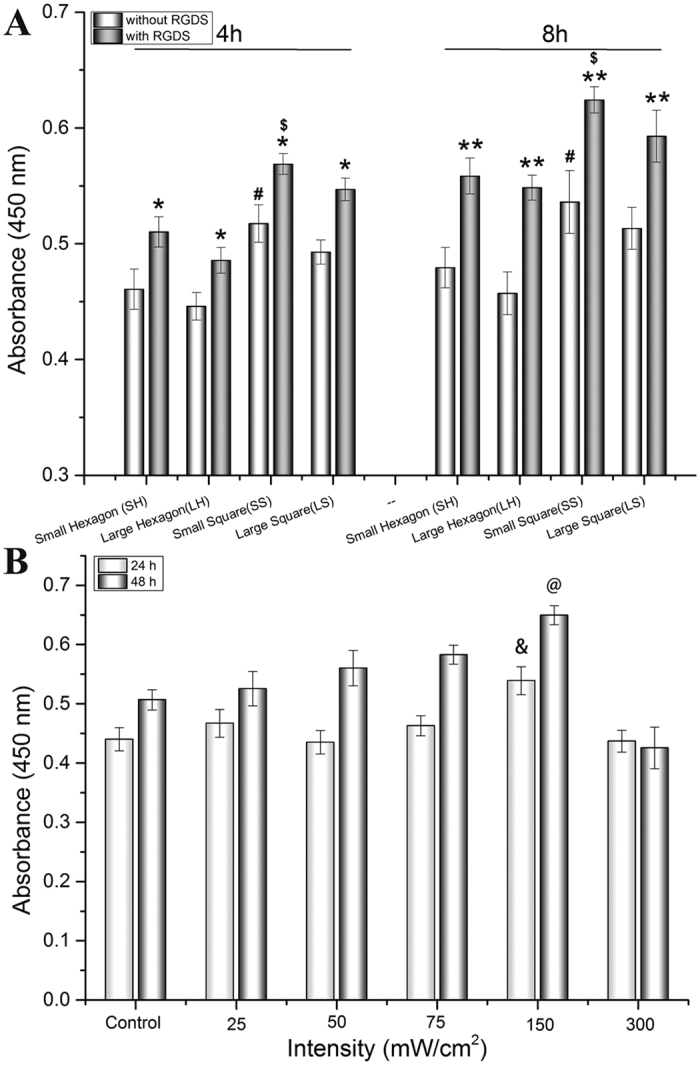
(**A**) 4 h and 8 h MSC adhesion on 3D printed scaffolds with and without RGDS, (**B**) 48 h MSC growth on the 2D culture plates under varied LIPUS treatment conditions. Data are mean ± standard error of the mean, n = 9. *p < 0.05, **p < 0.01 when compared to corresponding scaffolds without RGDS in each group. ^$^p < 0.05 when compared to all other scaffolds with RGDS. ^#^p < 0.05 when compared to all other scaffolds without RGDS. ^&^p < 0.05, ^@^p < 0.01 when compared to all other groups with various LIPUS intensities.

In order to investigate how scaffold geometry influences cell behavior and obtain optimal scaffold geometry, MSC adhesion on 3D printed scaffolds with varied geometric patterns was evaluated. The results showed that scaffolds with RGDS exhibited greater MSC adhesion than those without RGDS (*p* < 0.05) with increases of 8.9~11% in 4 h and 15.5~20% in 8 h, respectively. Moreover, small square pore scaffolds having higher MSC adhesion both with and without RGDS (Fig. 3A) (*p* < 0.05). These phenomena indicate that RGDS peptide can improve initial cell adhesion due to efficient binding between peptide ligands and MSC membrane receptors^14^,^15^. The RGDS moiety is a common cell adherent tetrapeptide^14^, therefore, increasing sites of membrane binding on the biomaterial substrate. Furthermore, the effectiveness of the small square geometry on MSC adhesion and spreading is postulated to be a curvature-driven effect related to relative porosity and pore size. Small pore scaffolds exhibit a greater porosity when compared to large pore scaffold (mentioned above). J. Knychala^27^ and Rumpler^28^ have reported that geometrical features of 3D-printed matrices affect cell behavior (adhesion, proliferation, migration and growth) due to curvature-driven cell growth and extracellular matrix deposition in scaffold with various geometries. MSC initially and preferentially adhere to corners and grow faster on larger curvatures. A similar phenomenon has been observed in our studies where attribute to larges curvature on small square pattern than that of other geometric pattern. Therefore, the 3D printed small square pore scaffold was chosen for subsequent stem cell proliferation and differentiation studies.

### Determination of Optimal LIPUS Parameters for MSC Growth

Optimal LIPUS excitation was determined by evaluating MSC proliferation under various intensities (20, 50, 75, 150 and 300 mW/cm^2^) cultured on a 24-well plate. After 48 h culture, MSC proliferation was evaluated in response to 3 minutes of LIPUS exposure (Fig. 3B) with the greatest response at 150 mW/cm^2^ when compared to the other intensities (*p* < 0.05). The highest MSC proliferation was observed after 24 h and 48 h under the LIPUS excitation with parameters of 1.5 MHz, 20% duty cycle and 150 mW/cm^2^ intensity. Comparable studies have reported that therapeutic ultrasound with intensities ranging from 15 to 300 mW/cm^2^ could facilitate bone fracture healing, bone union, tissue deposition and growth^19^,^24^,^25^. A low intensity of LIPUS may not induce sufficient mechanical stimulation for cell proliferation; while a high intensity of ultrasound may induce thermal degradation and cell lysis^26^,^29^. LIPUS stimulation of 150 mW/cm^2^ led to the promotion of MSC proliferation and was used for all experiments. Therefore, the work parameters of LIPUS stimulation of 1.5 MHz, 20% duty cycle and 150 mW/cm^2^ intensities was utilized and kept constant for osteogenic differentiation studies.

### The Effect of LIPUS Treatment on MSC Proliferation on 3D Printed Bioactive Scaffolds

**Figure 4:**
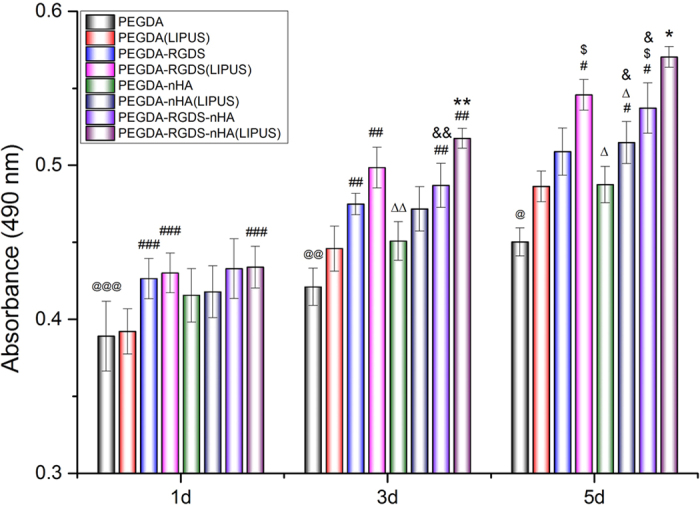
Enhanced MSC proliferation on 3D printed scaffolds with excitation after 1, 3 and 5 days of cultures. Data are mean ± standard error of the mean, n = 9. On day 1: ^@@@^p < 0.05 when compared to PEGDA-RGDS, PEGDA-RGDS-nHA and PEGDA-RGDS-nHA (LIPUS) groups; ^###^p < 0.05 when compared to PEGDA-RGDS, PEGDA-RGDS (LIPUS) and PEGDA-RGDS-nHA (LIPUS) groups. On day 3: ^@@^p < 0.05 when compared to all other groups except PEGDA (LIPUS) group; ^##^p < 0.05 when compared to PEGDA (LIPUS) group; ΔΔp < 0.05 when compared to PEGDA-RGDS (LIPUS) group; ^&&^p < 0.05 when compared to PEGDA-nHA group; **p < 0.05 when compared to all the other groups except PEGDA-RGDS (LIPUS) group. On day 5: ^@^*p* < 0.05 when compared to all other groups; ^#^*p* < 0.05 when compared to PEGDA (LIPUS) group; ^$^*p* < 0.05 when compared to PEGDA-RGDS group; Δ*p* < 0.05 when compared to PEGDA-RGDS (LIPUS) group; ^&^*p* < 0.05 when compared to PEGDA-nHA group; *p < 0.05 when compared to all other groups.

The effect of LIPUS treatment on MSC seeded on 3D printed scaffolds was investigated by evaluating MSC proliferation for 1, 3 and 5 days. At predetermined time points, cell proliferation was measured by MTS assay with the results shown in Fig. 4. MSC number increased in all groups with or without LIPUS excitation, however, an increase of 5.6–8% in cell density (*p* < 0.05) was observed in all groups with LIPUS treatment compared to untreated samples after 5 days culture. Cell proliferation on the PEGDA-RGDS-nHA group with LIPUS was the highest when compared to all other sample groups after 5 days (increasing up to 26.7%) (*p* < 0.05), as well as after 3 days treatment (increasing up to 22.9%) (*p* < 0.05). This increase in cell proliferation can be attributed to mechanical stimulation of adhered MSC when exposed to LIPUS excitation^30^. Wang *et al*. found increased MSC adhesion when cultured upon RGD-modified chitosan scaffolds illustrating the beneficial synergistic effects of LIPUS and bioactive scaffolds^31^. Furthermore, these results demonstrate the effectiveness of nHA as an additional bioactive promoter of desired MSC behavior. nHA is the most abundant inorganic bioactive and biocompatible constituent of bone commonly used in the fabrication of artificial orthopedic and dental implant^32^. It is a multifaceted nanomaterial promoting cell adhesion and proliferation^33^,^34^. In this work, MSC proliferation on scaffolds containing both nHA and RGDS was higher than bare PEGDA controls, and those containing only nHA or RGDS, respectively.

**Figure 5:**
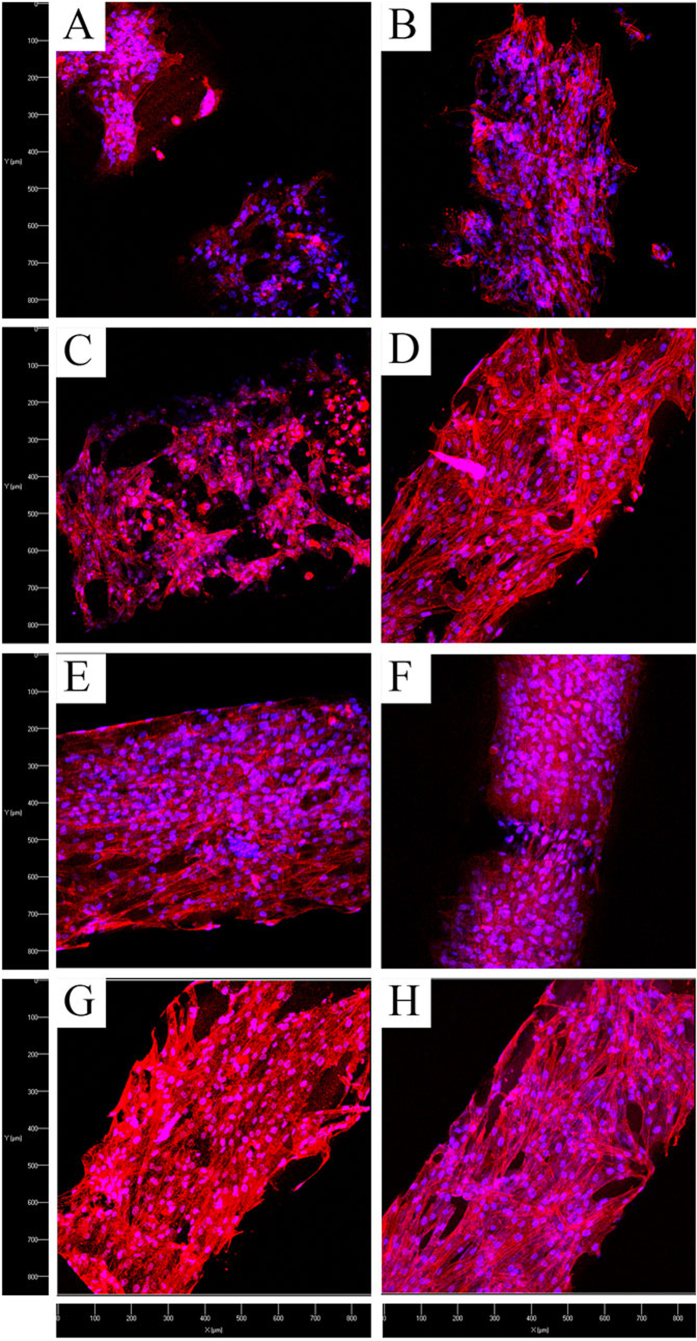
Confocal microscopy images of MSC proliferation on 3D printed scaffolds with and without LIPUS treatment after 5 days culture. (**A**) PEGDA without and (**B**) with LIPUS; (**C**) PEGDA-RGDS without and (**D**) with LIPUS; (**E**) PEGDA-nHA without and (**F**) with LIPUS; and (**G**) PEGDA-RGDS–nHA without and (**H**) with LIPUS, respectively. The cytoskeleton and cell nuclei were stained by Texas Red^®^-X phalloidin (red) and DAPI (blue), respectively.

Confocal microscopy analysis (Fig. 5) of MSC after 5 day culture illustrates excellent MSC attachment, spreading, and distribution on all scaffolds with or without LIPUS excitation. Notwithstanding, LIPUS treatment exhibited additional beneficial effects (Fig. 5(B,D,F,H)). Specifically, MSC display visible filopodia growth and extension on PEGDA-RGDS scaffolds treated with LIPUS (Fig. 5D). In addition, MSC proliferation rate was expedited to the extent that cell clusters occupied most of the pores of PEGDA-RGDS-nHA scaffolds after LIPUS treatment (Fig. 5H). Confocal microscopy analysis demonstrated that MSC strongly attached and proliferated on bioactive scaffolds with RGDS and nHA, especially, when treated with LIPUS for improved MSC cell growth.

### The Effect of LIPUS Treatments on MSC Osteogenic Differentiation on 3D Printed Scaffolds

**Figure 6:**
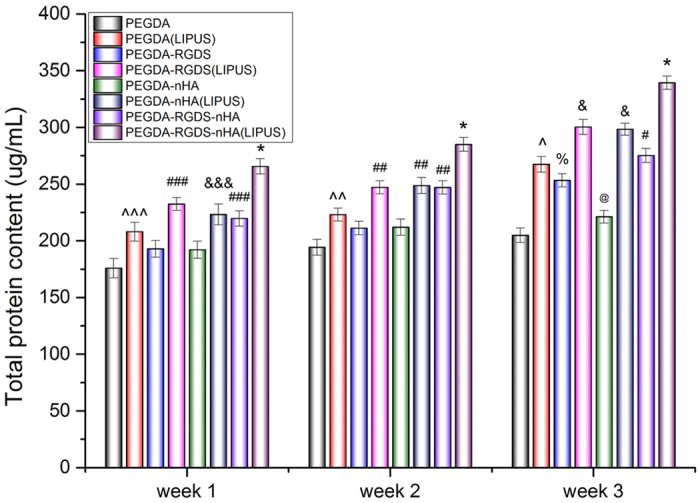
Improved total protein content on 3D printed bioactive scaffolds with LIPUS treatment after three-week MSC osteogenic differentiation. Data are mean ± standard error of the mean, n = 6. *p < 0.05 when compared to all other groups at respective weeks. Week 1: ^###^*p* < 0.05 when compared to PEGDA, PEGDA-RGDS and PEGDA-nHA; ^&&&^*p* < 0.05 when compared to PEGDA and PEGDA-nHA; and ^^^p < 0.05 when compared to the PEGDA group. Week 2: ^##^*p* < 0.05 when compared to all other groups and ^^p < 0.01 when compared to PEGDA. Week 3: ^&^*p* < 0.05 when compared to all other groups; ^#^*p* < 0.05 when compared to all other groups except PEGDA (LIPUS); ^*p* < 0.05 when compared to all other scaffolds except PEGDA-RGDS and PEGDA-RGDS-nHA; ^%^*p* < 0.05 when compared to PEGDA and PEGDA-nHA; ^@^*p* < 0.05 when compared PEGDA control.

**Figure 7:**
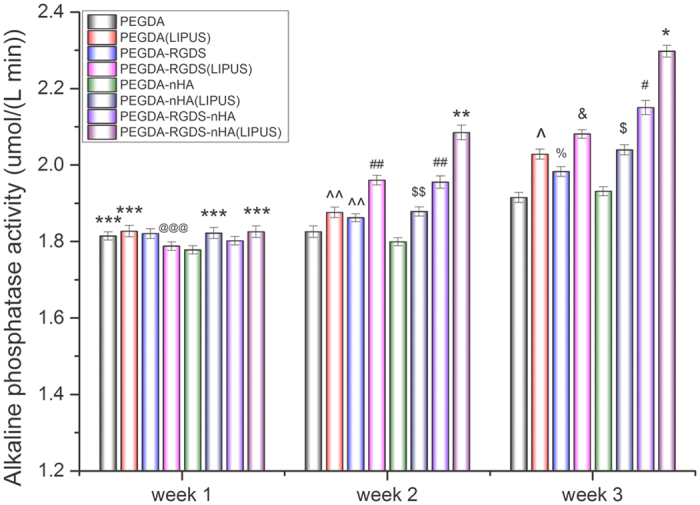
Greatly enhanced alkaline phosphatase activity on 3D printed bioactive scaffolds with LIPUS treatment after three-week MSC osteogenic differentiation. Data are mean ± standard error of the mean, n = 6. Week 1: ***p < 0.05 when compared to PEGDA-nHA; ^@@@^*p* < 0.05 when compared to PEGDA (LIPUS) and PEGDA-RGDS. Week 2: **p < 0.05 and ^##^p < 0.05 when compared to all other groups; ^$$^*p* < 0.05 when compared to all other groups except PEGDA (LIPUS) and PEGDA-RGDS; ^^*p* < 0.05 when compared to PEGDA-nHA. Week 3: *p < 0.05 and ^#^*p* < 0.05 when compared to all other groups; ^$^*p* < 0.05 when compared to PEGDA; PEGDA-RGDS and PEGDA-nHA; ^&^*p* < 0.05 and ^*p* < 0.05 when compared to all othe scaffolds except PEGDA-nHA (LIPUS); ^%^*p* < 0.05 when compared to PEGDA and PEGDA-nHA.

**Figure 8:**
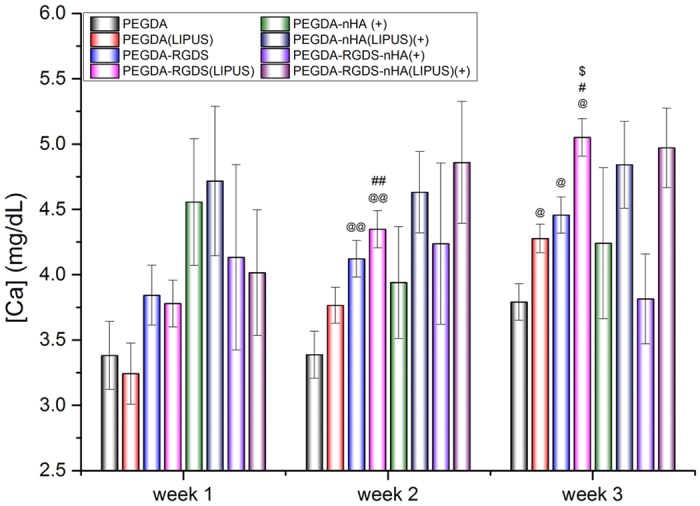
Calcium deposition on 3D printed bioactive scaffolds with and without LIPUS treatment after three-week MSC osteogenic differentiation. Data are mean ± standard error of the mean, n = 6. Week 2: ^@@^*p* < 0.05 when compared to PEGDA groups; ^##^*p* < 0.05 when compared to PEGDA (LIPUS) group. Week 3: ^@^*p* < 0.05 when compared to PEGDA groups; ^#^*p* < 0.05 when compared to PEGDA (LIPUS) group; ^$^*p* < 0.05 when compared to PEGDA-RGDS group. (+) symbol means: the calcium content in nHA had been subtracted from PEGDA-nHA and PEGDA-RGDS-nHA groups.

Osteogenic differentiation of MSC is an important phase in osseous tissue formation which is modulated by a myriad of factors including: genetic, metabolic, chemical, and mechanical stimulation^35^,^36^. Three-week osteogenic differentiation of MSC seeded on 3D printed bioactive scaffolds with or without LIPUS treatment was conducted. At predetermined time points, MSC were evaluated for total protein content, alkaline phosphatase (ALP) activity, and calcium deposition. The results of three-week MSC osteogenic differentiation are shown in Figs 6, 7, 8.

Total protein content of MSC increased over the course of this study with or without LIPUS treatment (Fig. 6). When compared to samples in the absence of LIPUS, the total protein levels in all LIPUS groups were significantly enhanced after 2 weeks by 14.9% (PEGDA), 17.0% (PEGDA-RGDS), 17.3% (PEGDA-nHA) and 15.4% (PEGDA-RGDS-nHA), respectively (*p* < 0.05). After 3 weeks, moreover, LIPUS groups increased by 30.6%, 18.6%, 34.9% and 23.3%, respectively, when compared to untreated groups (*p* < 0.01). In addition to the beneficial effects of LIPUS, significant increases in total protein were observed on RGDS/nHA-containing scaffolds after 3 weeks of culture. Of particular interest, PEGDA-RGDS-nHA scaffolds under LIPUS treatment exhibited the greatest total protein content when compared to all other groups. These results illustrate the synergistic effects of LIPUS and 3D printed bioactive scaffolds for improved osteogenic differentiation of MSC.

In addition, ALP is an early-stage marker of osteogenic differentiation as well as a precursor to calcium deposition. After 3 weeks of culture, ALP activity in all test groups increased over time (Fig. 7). When compared to samples in the absence of LIPUS excitation, ALP activity in all LIPUS groups (except for PEGDA group) was increased after 2 weeks and increased by 5.2% (PEGDA-RGDS), 4.4% (PEGDA-nHA) and 6.6% (PEGDA-RGDS-nHA), respectively (*p* < 0.01). Furthermore, greater expression was observed amongst all LIPUS groups after 3 weeks when compared to untreated groups. After 3 weeks, ALP activity in all LIPUS groups increased by 5.9% (PEGDA), 5.0% (PEGDA-RGDS), 5.6% (PEGDA-nHA) and 6.8% (PEGDA-RGDS-nHA), respectively, when compared to groups without LIPUS treatment (*p* < 0.01). These results show that LIPUS treatment can promote MSC proliferation and osteogenic differentiation. In addition, it is evident that RGDS and/or nHA containing scaffolds alone can elicit increased ALP activity when compared to bare PEGDA control.

Extracellular calcium deposition is a late-stage marker of osteogenic differentiation. Calcium deposition upon 3D printed scaffolds are shown in Fig. 8. Groups without nHA increased after 3 weeks of culture. In addition, in the absence of nHA, after 3 weeks, calcium deposition for LIPUS treated groups increased by 12.8% (PEGDA) and 13.3% (PEGDA-RGDS) (*p* < 0.05) when compared to samples without LIPUS treatment. These results demonstrate that calcium deposition on 3D printed bioactive scaffolds can be promoted by LIPUS stimulation. Extracellular calcium deposition amongst PEGDA-nHA and PEGDA-RGDS-nHA groups with and without LIPUS treatment were not statistically different after normalization by removal of incorporated nHA. With the subtraction of incorporated nHA (10% nHA), extracellar calcium deposition level on scaffolds was too small to detect when compared to within-group error in scaffolds and did not show statistical differences. Notwithstanding, when taken collectively, total protein and ALP activity results demonstrate the effectiveness of LIPUS excitation in promoting osteogenic differentiation of MSC.

The exact fundamental mechanism of LIPUS-mediated cell behavior is still not fully understood. Current available LIPUS cell studies have shown that mechanical stimulation (tensile strain, shock wave or shear stress…) produced by LIPUS may contribute to regulate MSC behavior. These stimulations will activate multiple signaling pathways, including the RhoA-GTP, p-Erk1/2, p-FAK, p-MEK1/2, p-p38, p-Akt, p-IKKα/β, NF-kB, intracellular calcium, and others. These pathways have been directly or indirectly involved in the production and synthesis of mineralization, ossification, ALP activity, protein and DNA from cells. All of the probable processes will greatly improve regeneration and repairing of bone tissue^37^,^38^,^39^.

## Conclusions

In the current work, RGDS and/or nHA-containing PEGDA 3D scaffolds with various geometric patterns were successfully printed by our novel table-top stereolithography 3D printer. The pores and channels in all scaffolds were well defined under SEM examination with increases in tensile strength for scaffolds containing nHA. Small square pores proved to be the optimal geometric pattern for MSC adhesion with optimal LIPUS excitation parameters of 1.5 MHz, 20% duty cycle with 150 mW/cm^2^ intensity. MSC proliferation and osteogenic differentiation demonstrate that RGDS and nHA readily enhance the bioactivity of PEGDA scaffolds. Additionally, our results also show that LIPUS can further promote MSC proliferation while enhancing osteogenic differentiation. Therefore, integrating LIPUS and 3D printing bioactive scaffolds can provide a valuable combinatiorial tool for improved MSC growth and osteogenic differentiation, thus making them promising for future regenerative medicine applications.

## Methods

### Preparation of 3D-printing bone scaffold based on stereolithograghy technology

3D printing hydrogel scaffolds were fabricated via a novel table-top stereolithography printer. Additional details about the stereolithography apparatus and the 3D printing process can be found here^5^. Prior to 3D printing, the 3D structural model was first designed by computer-aided design (CAD) software. Afterwards, the porosity, pattern of model and speed of laser were programmed via Slic3r computer numerical control conversion software. Briefly, CAD models were designed and printed layer-by-layer (Fig. 1). Due to the tenability of frequency of the pulsed signal (1~20 K Hz), the output laser energy can be controlled based the printing requirements of the bioink; and here we chose 8 K Hz for our study. In this study, we designed four models of varying geometries and pore sizes, including large square (LS) (500 μm), large hexagonal (LH) (500 μm), small square (SS) (250 μm) and small hexagonal (SH) (250 μm) (Fig. 2A). Three 400 μm thick layers were printed in approximately 2 square inches and an 8 mm biopsy punch was used to collect uniform, cylindrical scaffolds for cellular evaluation. The four test groups are: 60 w.t.% PEGDA + 40 w.t.% PEG +  photoiniator (0.5% of PEGDA mass) (PEGDA group); PEGDA group + 1 w.t.% Acryl-PEGDA-RGDS (PEGDA-RGDS group); PEGDA group + nHA (10 w.t. % of PEGDA mass)(PEGDA- nHA group); and PEGDA-RGDS group + nHA (10 w.t.% of PEGDA mass) (PEGDA-RGDS-nHA group).

Acryl-PEG-RGDS was synthesized according to Zhu’s method with slight modification^15^. Briefly, 1:1.2 M ratio of RGDS peptide (Arg-Gly-Asp-Ser) (American Peptide Company) and Acryl-PEG-NHS (JenKem Technology) were fully mixed and stirred overnight at 4 °C. Upon completion, the mixture was purified by dialysis (Slide-A-Lyzer™ Dialysis Cassettes, 3.5 K MWCO) to obtain the final Acryl-PEG-RGDS polymer.

nHA was prepared and characterized as described previously^6^. In brief, 0.6 M ammonium phosphate was dissolved in 825 mL pure water and the pH was adjusted to 9.0 by adding 50 mL ammonium hydroxide. 90 mL of 0.883 M calcium nitrate was added drop-wise at a rate of 3.6 mL/min under continuous stirring. The mixture was transferred into a Teflon liner and reacted hydrothermally at 200 °C for 20 h. Finally, nHA with a grain size of ~25 nm in width and 50–100 nm in length was obtained.

### Characterization

The porosity and morphology of samples was evaluated by scanning electron microscopy (SEM) (Zeiss Nvision 40FIB). Ultimate tensile strength of scaffolds was conducted via a mechanical testing machine (Applied Test Systems, Butler, PA). Briefly, scaffolds were cut into a strip with dimension of 10 mm × 30 mm and then fixed on the clips of the machine. The Young’s modulus will be recorded at speed of 1 mm/min (n = 6). Surface charge of the scaffold was measured by drop shape analysis using a contact angle analyzer (DSA4, Kruss).

### Cell culture

Primary human bone marrow MSCs were obtained from healthy consenting donors at Tulane University under an IRB-approved protocol with written informed consent. The Texas A&M Health Science Center, Institute for Regenerative Medicine further distributed the thoroughly characterized cells to us. We had the fully executed Material Transfer Agreement that was needed to obtain the cells. All experiments were performed in accordance with Materials Transfer Agreement. MSC (passage 3–6) were cultured in α-Minimum Essential Medium Eagle (α-MEM) supplemented with fetal bovine serum (FBS) (16.5%, v/v), penicillin and streptomycin (1%, v/v) and L-glutamine (1%, v/v). Cells were incubated at a condition of 37 °C, 5% CO_2_ and 95% relative humidity (RH). For MSC osteogenic differentiation, MSC were cultured in Dulbecco’s Modified Eagle’s Medium (DMEM) supplemented with FBS (10%, v/v), penicillin and streptomycin (1%, v/v), L-ascorbate acid (50 μg/mL), β-glycerophosphate (10 mmol/L) and dexamethasone (10 nmol/L). Cells were cultured as previously described.

### Optimization of the geometric pattern by MSC adhesion assay

Firstly, in order to obtain the optimal scaffold geometry (SH, LH, SS, LS), MSC adhesion was evaluated as a function of pore size and geometry. Prior to testing, scaffold samples were collected with a biopsy punch and immersed in 75% ethanol for 12 h. Afterwards, samples were placed in a 48-well plate followed by soaking in PBS and α-MEM for 12 h, respectively. Subsequently, MSCs were seeded upon the scaffold at a density of 5 × 10^4^ cells per cm^2^ and cultured for 4 h and 8 h. At predetermined time points, seeded scaffolds were washed three times with PBS to remove the non-adhered cells and the number of adherent cell was measured by Cell Counting Kit-8 (CCK-8) (Dojindo, Japan). Briefly, 10% reagent was added into a well plate followed by 2 h incubation and the absorbance was measured by a spectrophotometer at 450 nm (Thermo, USA). The results demonstrated that a scaffold with small square pores has greater MSC adhesion with or without RGDS (Fig. 3A). As a result, scaffolds with small square pores were used in all subsequent experiments.

### Ultrasonic device description and characterization

The tunable LIPUS system consists of a function generator, power amplifier and ultrasonic transducer. The ultrasound was produced by the function generator and amplified through a high power amplifier at a constant gain and emitted from the transducer. For operation in this study, the transducer was placed and oriented perpendicularly to the cell culture plate. To prevent bubbles from becoming trapped between the transducer face and the media, plates were filled to the brim. The transducer was then lowered into the well until a working distance of ~15 mm from the cell culture surface was achieved. Excitation was administered and the transducer was then removed, wiped with 70% ethanol, and the process repeated. Control samples were subjected only to submersion and withdrawal of the transducer without ultrasonic excitation.

### Optimization of the work parameter of LIPUS by 2D cell culture assay

Initially, MSC response to LIPUS was evaluated at 1.5 MHz, 20% duty cycle with various intensities (20, 50, 75, 150 and 300 mW/cm^2^). MSC were cultured on 24 well plates at 2 × 10^4^ cells per cm^2^ overnight. Next, the media was replaced to remove non-adherent cells and samples were subjected to ultrasonic excitation for 5 min. After 24 h and 48 h, the cell number was quantified via a CCK-8 assay and was compared to controls. The results demonstrated that MSC adhesion was greatest under 150 mW/cm^2^ (Fig. 3B). As a result, 1.5 MHz, 20% duty cycle with 150 mW/cm^2^ LIPUS excitation was used for all experiments.

### MSC proliferation on 3D-printing scaffold under LIPUS treatments

The effect of LIPUS on MSC proliferation seeded upon 3D printed bioactive scaffolds was evaluated. MSC (passage 3 to 6) were cultured in a 24-well cell culture plate. Briefly, four groups were tested (PEGDA, PEGDA-RGDS, PEGDA-nHA and PEGDA-nHA-RGDS) with small square pores were printed by our stereolithography-based 3D-printer as described above. Scaffolds measuring 15.5 mm in diameter were placed in 24-well plates followed by soaking in PBS and α-MEM for 12 h, respectively. Subsequently, MSC were seeded on the scaffolds at a density of 5 × 10^4^ cells per cm^2^ and cultured for 24 h. Afterwards, the media was replaced to remove non-adherent cells and the scaffolds were exposed to LIPUS excitation at 1.5 MHz, 20% duty cycle, 150 mW/cm^2^ intensities for 5 min every day. Cells were lifted and quantified as described above after 1, 3 and 5 days post-LIPUS treatment.

MSC morphology after 5 days of culture and LIPUS excitation was examined by laser confocal microscopy (Carl Zeiss LSM 710). All scaffolds were washed 3 times with PBS and fixed with 10% Formalin for 10 min followed by permeabilization in 0.1% Triton-100 for 10 min. MSC were double-stained with Texas red for 15 min, and 4′,6-diamidino-2-phenylindole (DAPI) for 3 min.

### MSC osteogenic differentiationof 3D culture under LIPUS treatments

A three-week osteogenic differentiation study was performed to evaluate the effectiveness of ultrasonic excitation on MSC osteogenesis. MSC were seeded at a density of 1 × 10^5^ cells per cm^2^ and cultured for 24 h. Non-adherent cells were removed and scaffolds were replenished with osteogenic differentiation media then subjected to LIPUS excitation at 1.5 MHz, 20% duty cycle, 150 mW/cm^2^ for 5 min every day. After 1, 2, and 3 weeks, the sample scaffolds were rinsed three times with pure water. Samples were lysed via freeze-thaw cycling (3×) and evaluated for total protein, alkaline phosphatase activity, and total calcium synthesis.

Total protein content of lysed samples was determined by a commercial BCA^TM^ Protein Assay Reagent kit (Pierce Biotechnology) according to the manufactures instructions. Briefly, 50 μL of each sample was loaded into a microplate well, then 150 μL of protein detection reagent was added, and incubated at 37 °C for 2 h. Absorbance of each sample was then measured at 562 nm and compared against a BSA standard curve to obtain total protein.

Alkaline phosphatase activity is one of the primary early indicators of osteogenic phenotype. The activity of lysed samples was determined by QuantiChrom^TM^ alkaline phosphatase assay kit according to the manufactures’ instructions. Briefly, a standard curve was created and run in parallel with lysed samples to determine the absolute concentration of alkaline phosphatase. 50 μL samples of lysate was added to a 96-well plate, followed by the addition of 150 μL work solution (the ratio of assay buffer, Mg Acetate and *pnPP* was 200:5:2) and mixed. The absorbance of the mixture was read at 405 nm and again after 4 min. Finally, the alkaline phosphatase activity was calculated per the kits instructions.

Total calcium content is one of the primary late-stage markers used to determine the osteogenic character of bone marrow derived MSC. Soluble extracellular calcium content was quantified by a commercial calcium reagent kit (Pointe Scientific, Inc.). Briefly, scaffold were immersed in a 0.6 N hydrogen chloride (HCl) solution at 37 °C for 24 h. Afterwards, 50 μL of lysate was combined with the o-cresolphthale in complexone solution to form a purple tinted solution. Solutions of known calcium concentration were run in parallel with the experimental group. Absorbance was measured spectrophotometercally at 570 nm.

### Statistical analysis

Data are presented as mean ± standard error of the mean and analyzed by one-way ANOVA. A p < 0.05 was taken as statistically significant.

## Additional Information

**How to cite this article**: Zhou, X. *et al*. Improved Human Bone Marrow Mesenchymal Stem Cell Osteogenesis in 3D Bioprinted Tissue Scaffolds with Low Intensity Pulsed Ultrasound Stimulation. *Sci. Rep.* **6**, 32876; doi: 10.1038/srep32876 (2016).

## Supplementary Material

Supplementary Information

## Figures and Tables

**Table 1:** The tensile modulus of 3D printed scaffolds.

Group	Young’s modulus of scaffolds (MPa)
PEGDA (LH)	0.839 ± 0.21
PEGDA (SH)	1.259 ± 0.197
PEGDA (LS)	1.425 ± 0.194^$^
PEGDA (SS)	2.016 ± 0.213^#^
PEGDA (SS)-RGDS	2.069 ± 0.206^#^
PEGDA (SS)-nHA	2.676 ± 0.194^*^
PEGDA (SS)-RGDS-nHA	2.633 ± 0.218^*^

LH: large hexagon; SH: small hexagon; LS: large square; and SS: small square. Data are mean ± standard error of the mean, n = 9. *p < 0.05, when compared to all other scaffolds. ^#^*p* < 0.05, when compared to PEGDA (LH/SH/LS) scaffolds. ^$^*p* < 0.05, when compared to the PEGDA (LH) scaffold.
