# Supplementary material for: Improved Human Bone Marrow Mesenchymal Stem Cell Osteogenesis in 3D Bioprinted Tissue Scaffolds with Low Intensity Pulsed Ultrasound Stimulation
Source: Sci Rep. 2016 Sep 6;6:32876. doi: 10.1038/srep32876 (PMC5011779; doi:10.1038/srep32876)
Supplement: Supplementary Information [file srep32876-s1.doc]

**Improved Human Bone Marrow Mesenchymal Stem Cell Osteogenesis in 3D Bioprinted Tissue Scaffolds with Low Intensity Pulsed Ultrasound Stimulation**

Xuan Zhou^1^, Nathan J. Castro^1^, Wei Zhu^1^, Haitao Cui^1^, Mitra Aliabouzar^1^, Kausik Sarkar^1^and Lijie Grace Zhang^1,2,3^*

1. Department of Mechanical and Aerospace Engineering, The George Washington University, Washington DC 20052, USA
2. Department of Biomedical Engineering, The George Washington University, Washington DC 20052, USA
3. Department of Medicine, The George Washington University, Washington DC 20052, USA

*Corresponding Author:

Dr. Lijie Grace Zhang

Tel: 202-994-2479

Fax: 202-994-0238

Email: lgzhang@gwu.edu

Mailing Address: 800 22nd Street NW Science and Engineering Hall, Room 3590, Washington DC, 20052


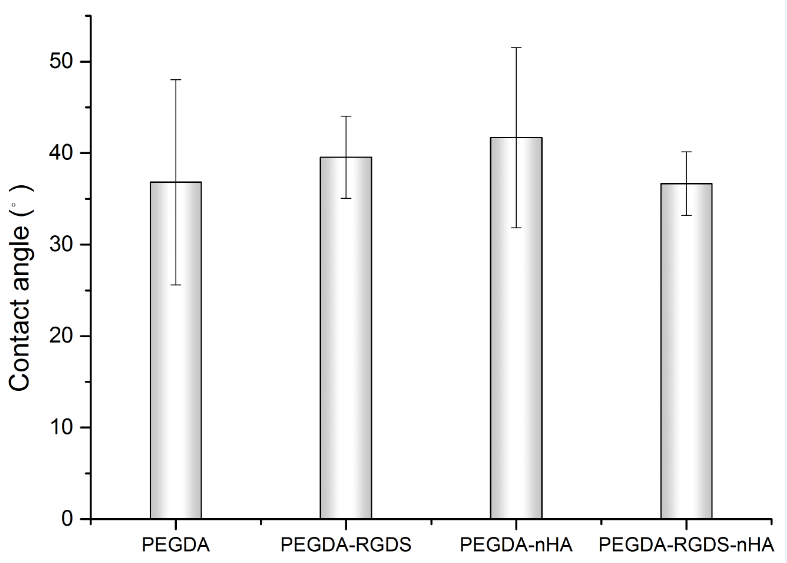


Figure S1. Contact angle of four scaffolds. Data are mean ± standard error of the mean, n=9.
